# Multiresistant Kawasaki Disease in a Young Infant with Giant Aneurysms Growing Fast

**DOI:** 10.3390/jcdd11050149

**Published:** 2024-05-14

**Authors:** Rosa Amorim-Figueiredo, Ana Pereira Lemos, Tiago Rito, Marta Conde, Maria João Brito, Fátima Pinto

**Affiliations:** 1 Pediatric Infectious Diseases Unit, Dona Estefânia Hospital, Unidade Local de Saúde São José, Academic Clinical Centre of Lisbon, 1169-045 Lisbon, Portugal; ana1645@gmail.com (A.P.L.); joao.rochabrito@netcabo.pt (M.J.B.); 2 Department of Pediatric Cardiology and Reference Center for Congenital Heart Diseases, Santa Marta Hospital, Unidade Local de Saúde São José, Academic Clinical Centre of Lisbon, 1169-024 Lisbon, Portugal; tiago.rito@gmail.com (T.R.); fatima.pinto@chlc.min-saude.pt (F.P.); 3 European Reference Network for Rare, Low Prevalence Complex Diseases of the Heart (ERN GUARD-Heart), 1169-024 Lisbon, Portugal; 4 Pediatric Rheumatology Unit, Dona Estefânia Hospital, Unidade Local de Saúde São José, Academic Clinical Centre of Lisbon, 1169-045 Lisbon, Portugal; marta.conde@chlc.min-saude.pt

**Keywords:** multiresistant Kawasaki disease, coronary aneurysms, intravenous immunoglobulin, anakinra, infliximab, propranolol

## Abstract

Background: Kawasaki disease (KD) is a type of vasculitis in which giant coronary artery aneurysms (CAAs) can occur. There are no specific guidelines for managing giant CAAs that develop quickly and are at risk of rupture. Regarding cardiovascular drugs, only beta-blockers are formally recommended in the acute phase of KD. Case presentation: A 6-month-old male patient with multiresistant Kawasaki disease and giant CAAs that continued to enlarge after controlling systemic inflammation was examined. The patient required three doses of intravenous immunoglobulin, methylprednisolone pulses, and anakinra and infliximab to normalize systemic inflammation. Due to the rapid increment of aneurysms’ dimensions and the risk of rupture, we introduced anticoagulant therapy and propranolol plus captopril, and titration doses were introduced according to a tolerated decrease in heart rate and arterial pressure. CAAs increment stabilized and slowly reduced their dimensions. Conclusions: The authors describe an atypical case of multiresistant KD with giant rapidly increasing CAAs even after controlling systemic inflammation. The introduction of a beta-blocker and an angiotensin-converting enzyme (ACE) inhibitor was demonstrated to be useful for stabilizing giant CAAs growth and reducing the potential risk of rupture.

## 1. Background

Kawasaki disease (KD) is the second most common vasculitis occurring in children worldwide, following Henoch–Schönlein Purpura [1]. It is a medium vessel vasculitis affecting coronary arteries [2], and it is currently the main cause of acquired heart disease in children of developed countries [3]. Coronary artery aneurysms (CAAs) develop in up to 25% of untreated cases [4], but giant CAAs are rare [5]. Early treatment with intravenous immunoglobulin (IVIg) decreases the incidence of CAAs to 5–7% [6]; nevertheless, 10–20% are refractory to treatment [7]. Treatment delay, age of less than one year, and refractory disease have shown to be associated with CAAs development [8]. Most coronary changes regress within five years, especially small aneurysms in children who have the disease before 12 months of age [5]. On the opposite end, giant aneurysms (≥8 mm in diameter or z-score of ≥10) rarely disappear, and they present an increased risk of myocardial infarction caused by occlusive thrombi [9].

Acute illness is treated with IVIg and a high dose of acetylsalicylic acid (ASA) [9]. Corticoid therapy is used in patients at a high risk of developing CAAs [9]. The treatment of refractory disease is controversial and depends on the experience of each team [10]. In case reports addressing giant CAAs, different approaches were taken [11,12]. 

Although biological drugs (such as anakinra and infliximab) have been shown to control giant aneurysms by reducing systemic inflammation [12,13], if giant CAAs continue to increase even after the resolution of fever and the normalization of inflammatory markers, cardiovascular drugs might have an important role in stabilizing CAAs growth. Guidelines to address giant aneurysms with rapid and decontrolled progression do not exist, and case reports published in the literature only focus on the use of biological drugs to treat these multiresistant cases. Regarding cardiovascular drugs, only beta-blockers are formally recommended by the American Heart Association (AHA) in the acute phase of KD [9], and to our knowledge, there are no case reports using angiotensin-converting enzyme (ACE) inhibitors together with beta-blockers in the acute phase of KD to prevent CAAs growth. 

Giant CAAs can rupture and cause a catastrophic event, so successful case reports are needed so that clinical trials can be made in order to establish specific guidelines addressing the situations in which giant CAAs continue to increase even after the resolution of fever and the normalization of inflammatory markers.

## 2. Case Presentation

**Figure 1:**
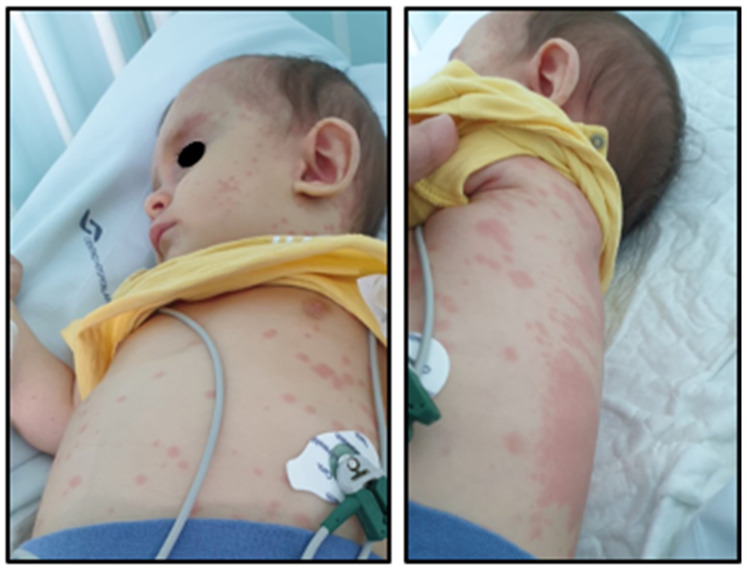
Maculopapular rash on day 2 of illness.

We present a case of a previously healthy 6-month-and-16-day-old male patient who was transferred to the Intensive Care Unit of our hospital on day 4 of illness due to shock in the context of high-grade fever; erythematous maculopapular rash affecting the face, trunk, palms, and soles (Figure 1); clefted lips; strawberry tongue; and diarrhea. Initial blood tests showed normocytic normochromic anemia (hemoglobin 11.3 g/dL), leukocytes 3300/uL with neutrophilia of 58.1%, platelets at 455,000/uL, an elevation of C-reactive protein (CRP) of 158.2 mg/L, erythrocyte sedimentation rate (ESR) of 85 mm/h, aspartate transaminase (AST) of 399 U/L, and alanine transaminase (ALT) of 393 U/L.

Repeated blood tests showed hemoglobin levels of 9.1 g/dL, CRP of 259.4 mg/L, procalcitonin of 51.12 ng/mL, and ESR of 75 mm/h. The lumbar puncture exhibited aseptic meningitis. Chest X-rays showed a bilateral reticular interstitial pattern with unilateral cisuritis. The echocardiogram was normal. The diagnosis of bacterial infection was considered, so ceftriaxone, clindamycin, and vancomycin were started.

The respiratory virus panel detected enteroviruses. Blood cultures; antigen stool tests for rotavirus, adenovirus, and norovirus; and PCR for SARS-CoV-2 on nasopharyngeal and oropharyngeal swabs were negative. Serologies for the Epstein–Barr virus, cytomegalovirus, parvovirus B19, human immunodeficiency virus, hepatitis A, *Mycoplasma pneumoniae*, *Rickettsia conorii*, *Leptospira interrogans*, and *Borrelia* spp. were also negative. Antistreptolysin O and anti-DNase B titers were negative.

**Figure 2:**
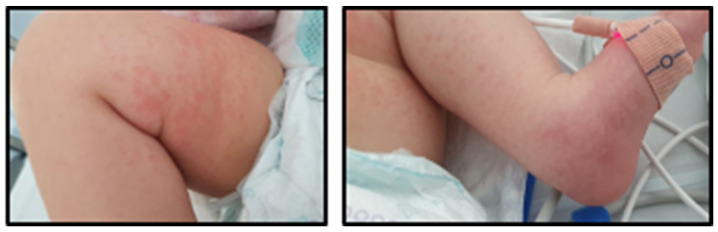
Macular rash on day 8 of illness.

The fever disappeared on day 5 of the illness, but mucocutaneous changes remained (Figure 2).

On day 7, normochromic normocytic anemia (hemoglobin level of 9.1 g/dL), hypoalbuminemia of 26.3 g/L, ALT elevation of 393 U/L, and sterile pyuria fulfilled the criteria to diagnose incomplete KD, presenting with shock. The echocardiogram remained normal until this time. The first dose of intravenous immunoglobulin IVIg 2 g/kg was administered. Treatment with methylprednisolone 2 mg/kg/day and oral lysine acetylsalicylate in a dose equivalent to 30 mg/kg/day of acetylsalicylic acid (ASA) was started. 

On day 8, albumin dropped to 19.2 g/L, so intravenous albumin 1 g/kg was administered. 

**Figure 3:**
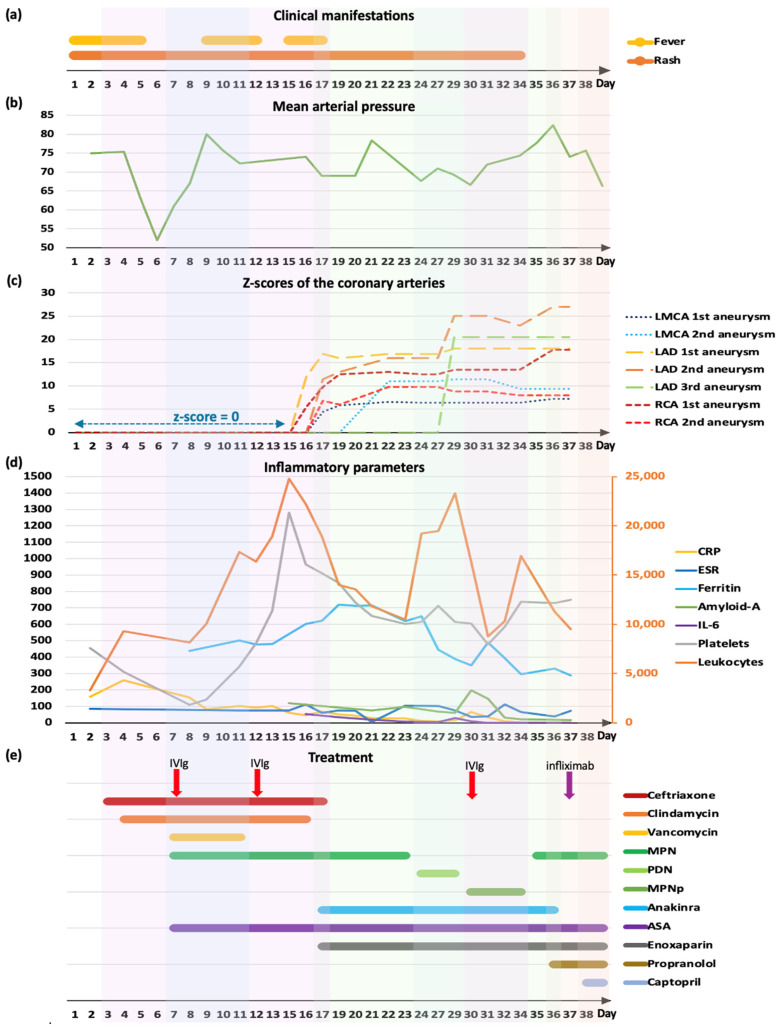
Clinical manifestations (**a**), mean arterial pressure (mmHg) (**b**), coronary arteries (**c**), inflammatory parameters (**d**), and treatment (**e**) according to the day of illness. Leukocytes (/uL) are shown in the right *y*-axis. The other inflammatory parameters are shown in the left *y*-axis. Amyloid-A (mg/L); ASA: acetylsalicylic acid; CRP: C-reactive protein (mg/L); ESR: erythrocyte sedimentation rate (mm/h); ferritin (ng/mL); IL-6: interleukin-6 (pg/mL); IVIg: intravenous immunoglobulin; LAD: left anterior descending artery; LMCA: left main coronary artery; MPN: methylprednisolone; MPNp: methylprednisolone pulses; PDN: prednisolone; platelets (×10^3^/uL); RCA: right coronary artery.

The fever returned on day 9, with blood pressure normalizing and the echocardiogram remaining normal (Figure 3). Considering refractory KD, a second dose of IVIg 2 g/kg was administered, with resolution of the fever for 2 days.

Alternative diagnoses were definitely ruled out by this time. Evidence of infection was no longer found. Blood cultures remained negative. The respiratory virus panel was repeated, and no viruses were detected. Stool PCR for enterovirus was negative, and no bacteria or parasites were found in stool analyses. MIS-C was also ruled out because the PCR for SARS-CoV-2 was negative on nasopharyngeal and oropharyngeal swabs, SARS-CoV-2 antibodies were not detected in blood, and the patient had not had close contact with a person with SARS-CoV-2 infection. Toxic shock syndrome was ruled out because diagnostic criteria were not fulfilled. Although soluble CD25 showed a high value of 20,500 pg/mL, the criteria for hemophagocytic lymphohistiocytosis were not fulfilled. The diagnosis of autoimmune diseases was unlikely since a low titer of antinuclear antibodies (ANA) of 1:80 was found, with a fine granular speckled pattern. 

Other types of vasculitis were also ruled out since serum complement levels were normal (C3 1.67 g/L, C4 0.19 g/L, and CH50 49 U/mL), serum cytoplasmic and perinuclear anti-neutrophil cytoplasm antibodies (ANCAs) were negative, and cerebral magnetic resonance angiography was normal. Adenosine deaminase 2 (ADA2) levels were normal, excluding their deficiency.

The fever reappeared on day 15. The echocardiogram performed on that day showed diffuse and irregular coronary dilatation for the first time. Giant CAAs appeared on day 16, with an echocardiogram showing a left anterior descending artery (LAD) with a maximum dilatation of 4.2 mm (z-score +12) and a right coronary artery (RCA) with a proximal dilatation of 3.1 mm (z-score +5.5) (Table 1). 

On day 17, anakinra was added to methylprednisolone, and enoxaparin in therapeutic dose was added to ASA. Anakinra was started on a low dose of 4 mg/kg/day on the first day and 6 mg/kg/day during the next 10 days. Antibiotic therapy was stopped on day 17. 

The patient had no more fever since anakinra was started, rash and palpebral edema improved, and inflammatory markers decreased (Figure 3). ASA was reduced to a low dose of 4 mg/kg/day, and IV methylprednisolone was switched to oral prednisolone. 

However, the echocardiogram performed on day 19 showed an escalation of the giant CAAs, which continued to increase with new areas of dilatation (Table 1). Despite this, inflammatory markers were decreasing, so treatment with anakinra was maintained.

While treatment continued with anakinra, inflammatory markers decreased for a few days without reaching a normal value and ended up increasing (Figure 3). On day 30 of illness, inflammatory markers were high, and the echocardiogram showed the enlargement of giant CAAs, so a third dose of IVIg 2 g/kg was administered and methylprednisolone pulses at 30 mg/kg/day were given daily for 5 days. The anakinra dose was also increased to 8 mg/kg/day.

By day 35 of illness, the patient remained without fever, a rash involution was observed, and inflammatory markers were normalized (Figure 3). Methylprednisolone was then kept at a low dose of 2 mg/kg/day.

However, coronary aneurysms continued to increase (Table 1). Therefore, we decided to stop anakinra and start propranolol at a low dose of 1 mg/kg/day 8/8 h, which was increased to a maximum dose of 2.5 mg/kg/day 3id. One single dose of infliximab 5 mg/kg was also administered. The patient was admitted to the cardiology unit for a close follow-up, and captopril 0.3 mg/kg/dose 3id was added. 

**Figure 4:**
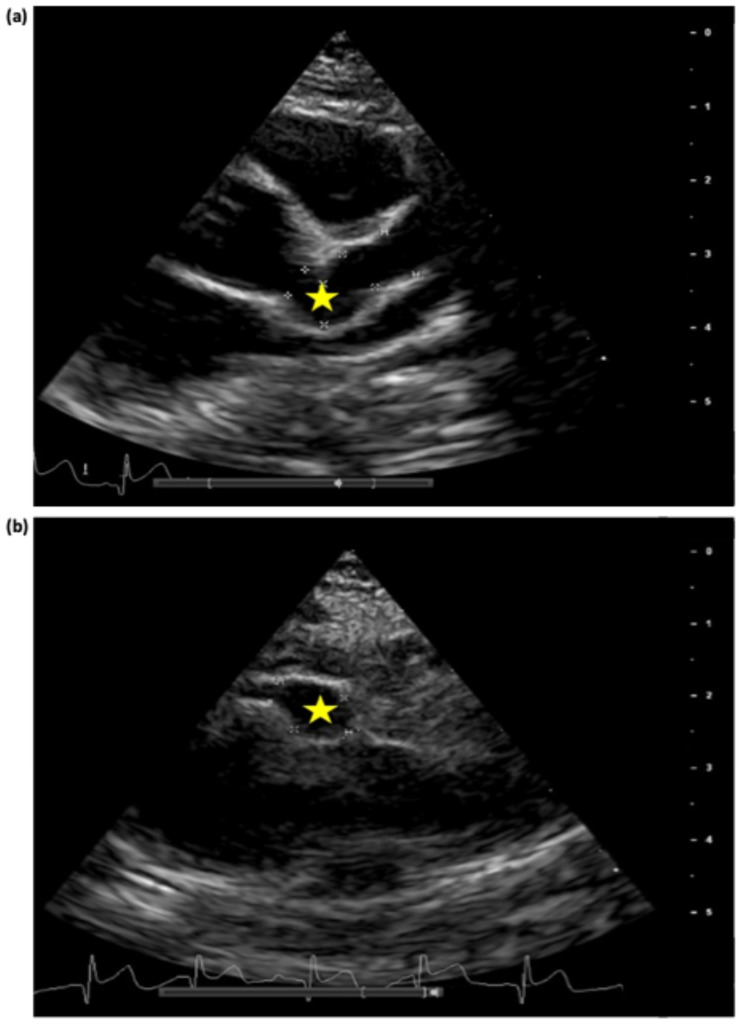
Echocardiogram performed on day 57 of illness, parasternal short axis view, showing left main coronary distal aneurysm with diameter of 5.5mm, z-score +10 (**a**), and right coronary aneurysm with diameter of 7mm, z-score +18.5 (**b**). The stars indicate the aneurysms. Scale in centimeters on the right-hand side of each image.

**Figure 5:**
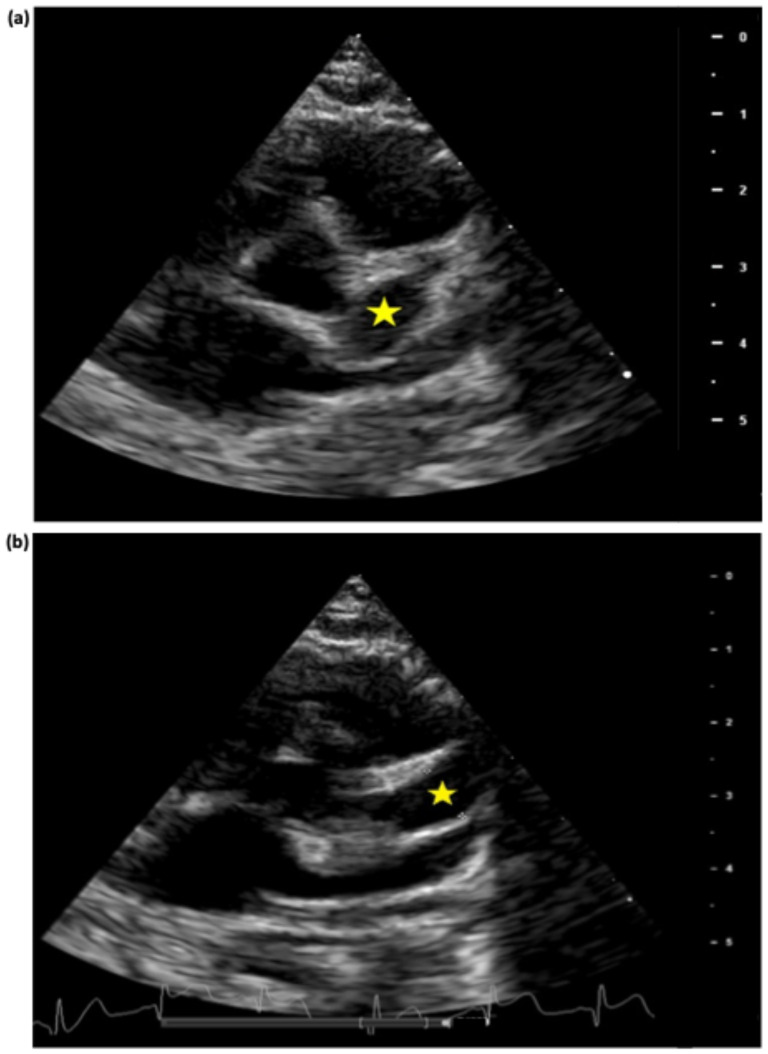
Echocardiogram performed on day 57 of illness, parasternal short axis view, showing left anterior descending artery proximal aneurysm with diameter of 5.5mm, z-score +18 (**a**), and left anterior descending artery distal aneurysm with diameter of 7.5mm, z-score +27 (**b**). The stars indicate the aneurysms. Scale in centimeters on the right-hand side of each image.

From day 36 of illness, stability of the giant CAAs was achieved (Figure 4 and Figure 5). Methylprednisolone was switched to prednisolone; oral warfarin was started, and enoxaparin was stopped when a therapeutic INR was reached.

The patient was discharged from the hospital on day 69 of illness. Prednisolone was discontinued after 4 weeks, but he remained in treatment with warfarin, AAS, propranolol, and captopril. Echocardiograms and coronary computed tomography angiogram performed after discharge showed that CAAs were regressing, and some of them had even disappeared, with LMCA, the distal part of LAD, and the distal part of RCA showing the resolution of aneurysms. Two aneurysms were still seen in the proximal and medium parts of LAD, with a maximum diameter of 4.5 mm (z-score +13.7), and one aneurysm was observed in the proximal part of RCA, with a maximum diameter of 3.4 mm (z-score +6.5) (Table 1). At 17 months of follow-up, the patient was asymptomatic and could easily climb stairs, and CAAs were decreasing, with LAD having only one proximal aneurysm of 3.6 mm (z-score +9.6) and RCA having only one proximal aneurysm of 3.5 mm (z-score +6.8), Table 1.

## 3. Discussion

We present a rare case of multiresistant KD with a rapid and uncontrolled development of giant coronary aneurysms that appeared 2–3 weeks after the onset of illness, albeit with early recognition and adequate management. Beyond the usual treatment of KD, a beta-blocker and an ACE inhibitor were necessary to stabilize CAAs progression. 

KD is considered a self-limited illness when coronary artery lesions do not occur [14,15]. When giant CAAs appear, a chronic vascular process begins [16]. The rapid dilatation of CAAs increases their risk of rupture, resulting in death [17].

Since specific guidelines addressing giant CAAs in KD are lacking, this successful case report of a patient with multiresistant KD and giant CAAs treated with two biological drugs (anakinra and infliximab) and two cardiovascular drugs (propranolol and captopril) can be useful so that specific guidelines can be made in the future.

Multiresistant KD is defined as the need for three or more drugs beyond IVIg to control acute inflammation [18]. The rate of resistance to a first dose of IVIg has increased over the years [18], and it is related to CAAs development [13].

The early recognition of KD can be difficult, since it can mimic several clinical entities, such as MIS-C, toxic shock syndrome, and sepsis. Sometimes, KD can have an atypical presentation, as it occurred with this patient, and alternative diseases have to be ruled out to establish the diagnosis of KD. In this patient, the diagnosis of septic shock caused by enterovirus or by a bacterium was considered at an initial stage. However, the appearance of coronary aneurysms confirmed the diagnosis of KD. 

KD shock syndrome rarely occurs in the acute phase of the disease, and it is commonly misdiagnosed as septic shock [19]. It is defined by systolic hypotension or clinical signs of poor perfusion in a patient with features of KD [19]. Its pathogenesis is not fully understood, and it is thought to be caused by capillary leakage related to vasculitis, cardiac systolic dysfunction, and inflammatory cytokine dysregulation [19]. Due to increased inflammation, these patients have a higher risk of IVIg resistance and CAAs development [19], so the first-line treatment includes corticotherapy beyond IVIg [20].

Coronary aneurysms arise in the first weeks after the beginning of KD, reaching their maximum by 6 weeks [21].

To reduce the likelihood of developing CAAs, IVIg must be administered within the first 10 days of the onset of symptoms and, if possible, within the first 7 days, which was what occurred in this case [22,23]. 

At admission, our patient presented with risk factors for IVIg resistance and, consequently, coronary aneurysms, such as an age of <12 months, hypoalbuminemia, elevated liver enzymes, decreasing hemoglobin levels, and elevated CRP [24]. Hypoalbuminemia has been shown to be an independent risk factor for the occurrence and progression of CAAs [25]. 

Considering that the risk of the rupture of a giant aneurysm mainly occurs in the first 2–3 weeks after the onset of fever [9] and in those with rapidly growing giant aneurysms, it is important to perform serial echocardiograms to detect, monitor, and promptly decide which pharmacological drugs should be started to prevent the progression of these aneurysms.

Giant CAAs are defined by a z-score of 10 or more, corrected for the body surface area, or by an absolute diameter of 8 mm or more [9]. Giant CAAs are related to a higher risk of myocardial infarction and sudden death, both in the acute phase of the disease and in the long term [9]. Coronary artery stenosis related to chronic inflammation mainly occurs in the first two years after the onset of the disease [26]. There are no specific guidelines for managing rapidly increasing giant aneurysms or even preventing their rupture in refractory KD patients. In order to control coronary artery vasculitis, several options are available after a second dose of IVIg, such as high-dose pulses of methylprednisolone and a third dose of IVIg, infliximab, anakinra, and immunosuppressors [9,27]. 

The combination of two biologicals consisting of a tumor necrosis factor (TNF)-α inhibitor and an interleukin-1 (IL-1) inhibitor had been previously shown to control aneurysm growth by reducing systemic inflammation in two KD patients aged six and three months [12,13]. 

Anakinra is an IL-1 receptor antagonist that has been shown to reduce coronary dilatation in an effective and safe way when used as a third-line drug [28,29,30]. This is why we chose anakinra as a third-line therapy for our patient. However, it was necessary to increase anakinra to the maximum dose of 8 mg/kg/day and add methylprednisolone pulses of 30 mg/kg/day and a third dose of IVIg to control the rash and inflammatory markers. Despite this, giant CAAs continued to increase.

Infliximab is a TNF-α inhibitor that has also been shown to be effective in controlling coronaries dilatation [31]. Therefore, one dose of infliximab was administered.

However, even after the apparent control of clinical and laboratory inflammation, coronary aneurysms continued to enlarge. The treatment with a beta-blocker and an ACE inhibitor was essential to control the aneurysm's diameter increase by promoting a mechanical and hemodynamic effect due to decreased heart contractility, heart rate, and blood pressure, avoiding the potential for continuous dilatation. 

The importance of reducing shear stress on the coronary endothelium and decreasing systemic pressure in order to prevent the further progression and rupture of aneurysms was previously shown by some KD case reports [32,33]. Although the evidence of using ACE inhibitors in the acute phase of KD is scarce, there is substantial experience with its use to control blood pressure in children. Most evidence regarding cardiovascular treatment in KD has been extrapolated from studies aiming at the long-term prevention of myocardial infarction in adults with atherosclerotic disease [9], and there are no guidelines to manage cardiovascular events associated with CAAs in the acute phase of KD.

Although AHA says that beta-blockers and ACE inhibitors might confer protection against myocardial infarction, only beta-blockers are formally advised for consideration when CAAs are present in KD [9]. The association of an ACE inhibitor with a beta-blocker initiated in the acute phase of KD in a normotensive infant aiming to prevent CAAs progression and rupture was not formally recommended but was suggested by some previous studies [21].

Beta-blockers inhibit the sympathetic system and were shown to have anti-inflammatory effects in atherosclerotic disease [34], and the reduction in myocardial oxygen consumption through beta-blockade was shown to prevent myocardial infarction in KD [35].

Vascular stenosis in KD occurs, at least in part, due to endothelial proliferation, in which the proinflammatory action of angiotensin II seems to play a role [34,36]. This is why ACE inhibitors might also be a good choice to control CAAs even in the acute phase of KD.

Our patient was discharged from the hospital nearly 2 months after admission. At this time, the aneurysms' diameters were stable. At 26 months old, after 17 months of follow-up, the patient was asymptomatic, and the CAAs diameter was progressively decreasing. Patients with CAAs require lifetime follow-ups in cardiology because their coronary structure and function remain affected even if CAAs dimensions return to normal, with the potential for future complications [9]. Risks of stenosis, occlusion, and the formation of new aneurysms remain throughout life, and an increased risk of atherosclerosis, although still controversial, adds an additional risk for cardiac events [9].

As long as CAAs remain, keeping the beta-blocker should be considered, since it seems to prevent myocardial ischemia [34,37]. Keeping the ACE inhibitor should also be considered, since it seems to prevent stenosis adjacent to the CAAs [34,37].

## 4. Conclusions

The authors present a successful case of multiresistant KD with giant CAAs in rapid progression even after controlling systemic inflammation, in which treatment with propranolol and captopril was essential to prevent the further dilatation and rupture of coronaries.

There are no specific guidelines to prevent rapidly increasing aneurysms and their rupture, so clinical trials are warranted to clarify the role of the combination of a beta-blocker and ACE inhibitor in the acute phase of KD.

## Figures and Tables

**Table 1:** Evolution of echocardiographic changes in coronary arteries.

	Infectiology Unit			Cardiology Unit		Follow-Up (Months after Discharge)		
	D16	D19	D29	D36	D69	1 Month	4 Months *	17 Months
LMCA	Ø	1st 4 mm (z-score +5.8)	1st 4.2 mm (z-score +6.4)	1st 4.5 mm (z-score +7.2)	1st 4 mm (z-score +5.8)	Ø	Ø	Ø
		Ø	2nd 6 mm (z-score +11.4)	2nd 5.3 mm (z-score +9.4)	2nd 5.5 mm (z-score +10)			
LAD	1st 4.2 mm (z-score +12)	1st 5 mm (z-score +16)	1st 5.5 mm (z-score +18)	1st 5.5 mm (z-score +18)	1st 5.5 mm (z-score +18)	1st 5.4 mm (z-score +17.8)	2 aneurysms max diameter 4.5 mm (z-score +13.7)	1st 3.6 mm (z-score +9.6)
	Ø	2nd 4.4 mm (z-score +13)	2nd 7 mm (z-score +25)	2nd 7.5 mm (z-score +27)	2nd 6.8 mm (z-score +24)	2nd 6.6 mm (z-score +23)		Ø
	Ø	Ø	3rd 6 mm (z-score +20.5)	3rd 6 mm (z-score +20.5)	Ø	Ø	Ø	Ø
RCA	1st 3.1 mm (z-score +5.5)	1st 5.2 mm (z-score +12.5)	1st 5.5 mm (z-score +13.5)	1st 6.8 mm (z-score +17.8)	1st 7 mm (z-score +18.5)	5.4 mm (z-score +13)	Max diameter 3.4 mm (z-score +6.5)	1st 3.5 mm (z-score +6.8)
	Ø	2nd 3.2 mm (z-score +6)	2nd 4.1 mm (z-score +8.8)	2nd 3.9 mm (z-score +8)	Ø	Ø	Ø	Ø

Boston z-scores are presented and calculated for the same weight and height seen at admission. Coronary aneurysms are numbered from the most proximal to the most distal. * Coronary computed tomography angiography was performed at 4 months after discharge. LAD: left anterior descending artery; LMCA: left main coronary artery; RCA: right coronary artery; Ø: no data.

## Data Availability

Further details of this case can be accessed by contacting any of the authors.

## Abbreviations

ACE	Angiotensin-converting enzyme
ADA2	Adenosine deaminase 2
AHA	American Heart Association
ANA	Antinuclear antibodies
ANCA	Anti-neutrophil cytoplasm antibody
aPTT	Activated partial thromboplastin time
ASA	Acetylsalicylic acid
ALT	Alanine transaminase
AST	Aspartate transaminase
CAA	Coronary artery aneurysm
CRP	C-reactive protein
CSF	Cerebrospinal fluid
DNA	Deoxyribonucleic acid
ESR	Erythrocyte sedimentation rate
IL-1	Interleukin-1
IL-6	Interleukin-6
INR	International normalized ratio
IVIg	Intravenous Immunoglobulin
KD	Kawasaki disease
LAD	Left anterior descending artery
LMCA	Left main coronary artery
MIS-C	Multisystem inflammatory syndrome in children
MPN	Methylprednisolone
MPNp	Methylprednisolone pulses
PCR	Polymerase chain reaction
PDN	Prednisolone
RCA	Right coronary artery
TNF	Tumor necrosis factor
